# Explicit Mentalizing Mechanisms and Their Adaptive Role in Memory Conformity

**DOI:** 10.1371/journal.pone.0062106

**Published:** 2013-04-18

**Authors:** Rebecca Wheeler, Kevin Allan, Dimitris Tsivilis, Douglas Martin, Fiona Gabbert

**Affiliations:** 1 School of Psychology, University of Aberdeen, Aberdeen, United Kingdom; 2 School of Psychological Sciences, University of Manchester, Manchester, United Kingdom; 3 Department of Psychology, Goldsmiths University of London, New Cross, London, United Kingdom; Okayama University, Japan

## Abstract

Memory conformity occurs when an individual endorses what other individuals remember about past events. Research on memory conformity is currently dominated by a ‘forensic’ perspective, which views the phenomenon as inherently undesirable. This is because conformity not only distorts the accuracy of an individual's memory, but also produces false corroboration between individuals, effects that act to undermine criminal justice systems. There is growing awareness, however, that memory conformity may be interpreted more generally as an adaptive social behavior regulated by explicit mentalizing mechanisms. Here, we provide novel evidence in support of this emerging alternative theoretical perspective. We carried out a memory conformity experiment which revealed that explicit belief-simulation (i.e. using one's own beliefs to model what other people believe) systematically biases conformity towards like-minded individuals, even when there is no objective evidence that they have a more accurate memory than dissimilar individuals. We suggest that this bias is functional, i.e. adaptive, to the extent that it fosters trust, and hence cooperation, between in-group versus out-group individuals. We conclude that memory conformity is, in more fundamental terms, a highly desirable product of explicit mentalizing mechanisms that promote adaptive forms of social learning and cooperation.

## Introduction

Mentalizing is the ability to understand the covert mental states that underlie one's own and other people's overt behavior. According to Frith and Frith [1], mentalizing serves two adaptive roles in social cognition. Mentalizing about another person's knowledge relative to one's own can bias social learning towards individuals offering complementary or superior knowledge, thus enhancing the accuracy of our own representations of reality [1] [2]. In addition, keeping track of other people's beliefs, desires and intentions in relation to one's own may generate trust, foster cooperation and promote joint endeavors, such as science, that indirectly benefit individuals [1]. We have recently argued [3] that the adaptive perspective on mentalizing may illuminate our understanding of memory function in social contexts, an area of research that is currently dominated by a ‘forensic’ perspective. We provided evidence [3] that memory conformity is regulated by explicit mentalizing mechanisms that promote accuracy in the precise adaptive manner described by Frith and Frith [1]. In this paper, we propose a further explicit mentalizing mechanism that could functionally bias conformity in the absence of objective evidence that one person's memory is superior to that of another. This proposal is tested in a novel experiment, which reveals that simulating another person's beliefs (i.e. using one's own beliefs as a model for their beliefs) biases individuals to conform to the memory of like-minded others who are similar to one's self.

The recent surge in research dealing with social influences upon memory was inspired by seminal studies, beginning in the 1970s, which examined the effect of accurate and inaccurate post-event information on eyewitness memory (reviewed in [4]). In this context, regardless of whether a person accepts accurate or inaccurate information from a co-witness, the resulting corroboration between their testimonies is false and therefore undesirable. From this forensic perspective, other people are merely the source of highly contagious contaminants that cause memory to malfunction [5], in the very specific sense that social influences can ‘corrupt’ the representation of what an individual perceived or thought during some event. With some justification then, research has focused on the nature of the memory distortions [6] and patterns of false corroboration [7] that result from conformity to another person's memory, as well as factors that promote or inhibit conformity (e.g. [8] [9] [10] [11] [12]). This research, however, has consistently revealed patterns of evidence suggesting that conformity is not dysfunctional. Instead, as noted above, conformity appears to be a strategic response guided by explicit mentalizing mechanisms that bias social learning to promote memory accuracy in the adaptive manner suggested by Frith and Frith [1] (for similar conclusions see [13] [14]).

Two complementary approaches are typically used to investigate how conformity covaries with the relative accuracy of memory in self versus other. These approaches either involve manipulating one's own memory accuracy, or manipulating what participants believe about the quality of their partners' memory. Using the former approach, studies have found that conformity increases when the accuracy of one's own memory decreases, and vice versa (e.g. [5] [15] [16]). Studies using the latter approach, i.e. examining whether conformity is a function of the perceived accuracy or credibility of a social partner, show that it increases as the likely accuracy or credibility of one's partner increases, and vice versa (e.g. [10] [11] [14] [17]). To bring these two approaches together, we proposed [3] that memory conformity involves a strategic trade-off that balances the substantial pros and cons involved when people learn from one another about the past [18] [19]. We reported [3] that although individuals did conform more when shorter encoding durations led their own memory to fail, this only occurred when individuals believed that the person they were collaborating with had been able to encode stimuli for a longer duration. That is, individuals supplemented their own worsening memory by increasingly relying on their partner's memory only when they had reason to trust in the accuracy of their partner's memory.

The studies described above show quite clearly that memory conformity involves the ability to evaluate communications from others regarding the content of their memory. In particular, to judge the relative reliability of another person's memory versus one's own memory, individuals appear to use their meta-cognitive knowledge about factors, such as encoding duration, that modulate the accuracy of memory. This corresponds precisely to the regulatory role that Frith and Frith [1] assign to meta-cognitive mechanisms during social interactions, based on their review of work on how people judge one another's perceptual abilities, cooperative (or deceptive) intent and agency. Studies of memory conformity are therefore beginning to illuminate how meta-cognition functions to regulate the accuracy of an individual's memory in specific social settings, a key issue highlighted for future research by Frith and Frith [1].

So far, however, we have considered only how the social application of meta-cognition may directly benefit individuals by regulating when and to whom they conform, based on considerations of memory accuracy in self vs. other. But Frith and Frith [1] also proposed that the ability to keep track of another person's beliefs in relation to one's own has a specific socially beneficial function. While fully acknowledging its Machiavellian ‘dark side’, Frith and Frith [1] point out that knowledge of other people's inner states can also promote trust and cooperation, which can produce indirect benefits for individuals themselves. Here, we hypothesize that a particular explicit mentalizing mechanism may play a key role in building trust between individuals, and to test our proposal we examine whether the engagement of this mechanism leads to significantly enhanced trust in another person's memory, even when there is no objective evidence that their memory accuracy is superior to that of another social partner. Our hypothesis is that explicit mentalizing builds trust by helping us to distinguish between like-minded, in-group, versus dissimilar, out-group, individuals.

Specifically, we propose that simulation-based mentalizing [20] [21] [22] is a mechanism that allows us to appreciate the degree of overlap between one's self and other people. Hence, we predict that increased belief-simulation during a ‘social inference’ task should enhance memory conformity to like-minded individuals who are similar to ones' self (for a related hypothesis on the effects of simulating motor-acts in others, see [23]). To test the prediction, a novel procedure (described below) was developed that integrates the virtual partner collaborative memory test [3] [8] with the virtual partner methodology developed by Mitchell and colleagues to investigate simulation-based mentalizing [20] [21].

First of all, participants were informed that they would get to know two strangers, based solely on their opinions about various issues taken from the set constructed by Mitchell, Macrae and Banaji [20]. Via computer, participants gave their own opinion, and then they saw the opinions of their two virtual partners. These were manipulated so that across a total of 20 exposure trials, one partner agreed 75% of the time with the participant, while the other partner disagreed 75% of the time. Following this exposure phase, participants then tried to infer their partners' opinions on a further series of novel issues. When this mentalizing phase was complete, participants next engaged in a collaborative memory test with their two partners. During this memory phase, participants viewed images of household scenes, and then performed the 2-alternative forced-choice memory test [3] [8]. Participants were told that they would view each partner's response before making their own, private, response. The partner responses during the memory test were controlled such that on a quarter of the trials they both endorsed the correct response option, and on a quarter of trials they both endorsed the incorrect response option. On the remaining half of the memory trials, the partner's responses disagreed with one another such that on a quarter of the trials only the similar partner was correct while on the other quarter only the dissimilar partner was correct. It is crucial to note that the frequency of correct and incorrect responses from each partner is therefore identical, and hence any systematic bias in conformity to one partner over the other cannot be due to objectively present accuracy differences.

After completing the collaborative memory test, each participant was finally asked to give their own opinion on those issues previously encountered during the mentalizing phase. To measure how the manipulation of agreement during the exposure phase alters explicit mentalizing, we aligned the participants' mentalizing phase responses with their own opinions as expressed in the final phase of the experiment. The resulting ‘mirror’ score allows us to quantify belief-simulation and establish whether it is enhanced towards the more agreeable (i.e. similar) versus less agreeable (i.e. dissimilar) partner. Our prediction is that using one's own views to simulate another person's – projecting one's own beliefs onto that person - will systematically bias to whom we subsequently choose to conform. As reported below, this was indeed the case.

## Materials and Methods

### Ethics Statement

The research was approved by the University of Aberdeen, School of Psychology Ethics Committee. Informed consent was obtained in writing from all participants prior to participation, and all participants were debriefed immediately after participation.

### Participants

102 undergraduate psychology students recruited in return for course credit (48 male, mean age 19.1, SD = 2.1) with each person attending one of four group sessions.

### Stimuli

We obtained the full set of opinion statements used by Mitchell et al. [20], adjusting their wording for the UK context and rejecting any that could not be so adjusted. Each of the remaining 190 statements was then independently rated, by 24 people, using a Likert scale. This asked raters to estimate how informative each statement was about a person's underlying character. We then ranked all the statements according to their mean rating. Then we took the top 30 most informative statements by rank and split these randomly into 3 sets of ten, equating the mean rating in each set. These sets were then counterbalanced across participants for viewing in either the exposure or the mentalizing phases described below. As filler items, we also used the 10 bottom ranking, i.e. least informative, opinion statements. To illustrate, the top 3 ranking opinion statements were: “I believe public education is a waste of time and would enroll my children in private school”; “I believe that eating meat is morally questionable”; “I am annoyed by spending time with people who have different opinions to me”. Whereas, the bottom 3 ranking opinion statements were: “I prefer red wine to white wine”; “I enjoy eating chicken soup”; “I enjoy listening to Radio”.

For the collaborative memory test we employed the household scenes first used by Roedier, Meade and Bergman [5] and subsequently by us [3] [8]. Each participant viewed 3 of these scenes, and we then tested memory for scene details using 90 2-alternative forced choice (2AFC) questions, with 30 questions from each scene. Within these 30 questions we randomly formed four sets of 7 questions to effect the counterbalancing of four social information conditions, of which two comprised conflicting information from each of the virtual partners (similar-accurate/dissimilar-errant vs. similar-errant/dissimilar-accurate) and two contained information from each partner that agreed upon a single response (accurate vs. errant). On the remaining two questions for each scene we provided no social information at all purely to enhance the realism of the virtual interaction in order to mimic trials where the partner's did not remember any information or were unable to respond quickly enough within a trial. To illustrate, participants were asked questions such as: “Was the Bathroom window open or closed?” (Response alternatives: “Open”/“Closed”); “What color was the kettle in the Kitchen?” (Response alternatives: “Black”/“Gray”); “What kind of fruit did you see in the bedroom?” (Response alternatives: “Apple”/“Banana”).

### Procedure

The experiment took place in a networked computer lab at which participants attended in groups of around 24 individuals. Participants were informed that we were investigating how people get to know one another based on what beliefs they hold. Then we informed the participants that they would be paired up randomly with two other anonymous individuals from their group and that each triad would interact via computer during the whole experiment. Participants were informed that to preserve anonymity throughout the experiment, one of their partners would be labeled as BEAR and the other as TIGER (note that the similar and dissimilar virtual partners were each given one of these names equally often across participants).

Exposure phase: participants were informed that the first part of the experiment involved exposure to their partners opinions about 20 different issues. As noted above, these consisted of a set of 10 highly informative statements along with the 10 filler items. Each statement was shown on screen, with two response options shown below (“AGREE” or “DISAGREE”). The participant was instructed to give their own response, self-paced, and then the responses of the two partners were given following a brief simulated reaction time (using values taken from a distribution with a mean of 3.0s and an SD of 300ms). This information was indicated by putting the partners name underneath either the AGREE or the DISAGREE response, thus signaling whether the partners agreed or disagreed with the participant's own response. The partners' responses were manipulated so that one partner agreed on 75% (15/20, of which 10 were highly informative statements and 5 were fillers) and the other partner disagreed on 75% of trials (15/20,10 highly informative and 5 fillers).

#### Mentalizing Phase

Immediately after the exposure phase, participants were told that we would now assess what they had learned about their partners by asking them to try and predict their partners' responses to a new set of opinion statements. We showed each of these 20 new statements, asking for either BEAR's (10 statements) or TIGER's (10 statements) predicted response. The order of BEAR/TIGER was randomized from trial to trial.

#### Memory Phase

Following the mentalizing task, we then informed the subjects that they and their partners would view a series of 3 household scenes for 2 minutes each. After this encoding phase we told the participants that they would now engage in a collaborative memory task with their partners, and that they would be able to view their partners' responses to each 2AFC question before giving their own private response that would not be viewable by either of their partners. We also told the participants that occasionally they might not see any responses if one or other of the partners did not respond within a 3s interval from the onset of the 2AFC question. We made the participants explicitly aware at this point that they were free to use the information from their partners as they saw fit, and that we would be assessing the accuracy of their memory as opposed to the overall accuracy of the triad. These instructions are therefore essentially identical to those employed in our prior studies using these stimulus materials and the 2AFC task [3] [8].

#### Mirror Phase

Finally, after the collaborative memory task was complete, we asked the participants to give us their own opinion on the 20 statements shown previously during the mentalizing task. In total, this whole procedure took ∼45minutes to complete.

## Results

### Simulation-Based Mentalizing Data

First, we examined the degree to which participants' own opinions during the final phase of the procedure mirrored those given during the mentalizing task that preceded the collaborative memory test. If responses during the mentalizing task were essentially random, i.e. they did not systematically derive from the participant's own opinions or their opposite, then the mirror scores should be at chance (i.e. 5 out of 10, given the binary ‘agree’ or ‘disagree’ response option available for each opinion statement). Against 5 as the null hypothesis value, however, one-sample t-tests revealed that the similar-partner mirror score of 7.61 (SD = 1.69) was significantly higher than chance (t(101) = 15.65, p<0.0001), whereas the dissimilar-partner mirror score of 5.08 (SD = 2.16) did not differ from chance (t(101) = 0.37, p = 0.72). Hence, as predicted, the mirror score was significantly higher for the similar vs. dissimilar partner (t(101) = 8.94, p<0.0001). These findings strongly suggest that the similarity manipulation enhanced simulation-based mentalizing towards the similar partner. Indeed, we could not find evidence for any simulation of the dissimilar partner.

### Memory Conformity Data

Having established that the similarity manipulation modulated the extent to which participants simulated their two partners in the desired direction, we now examine whether simulation biases the expression of conformity during the memory task. This was examined by contrasting the participants' 2AFC response accuracy in the conditions where they received conflicting social information from each partner about the past. If simulation based mentalizing systematically biases whom we express agreement with, participants' should conform more often to the responses of the more highly simulated partner and less often to the responses of the other partner. This should cause a pattern of enhanced and impaired 2AFC responses according to whether the similar partner's response was correct or incorrect, respectively. Alternatively, if simulation fails to bias how participants react to social information about the past, then we should observe no systematic difference in 2AFC performance according to the accuracy of the similar partner. What we observed was that when the similar partner was correct (and the dissimilar partner was therefore incorrect), the mean correct 2AFC rate was 76.6% (SD = 12.1). But when the dissimilar partner was correct (and the similar partner was therefore incorrect) the mean correct 2AFC rate dropped to 70.1% (13.7). This difference was significant (t(101) = 3.66, p<0.001). That is, participants did in fact conform significantly more often to their similar versus dissimilar partner when faced with conflicting social information about the content of the scenes they had encoded.

We also examined the difference in correct 2AFC performance between the conditions where each partners' responses agreed on the correct versus incorrect options. When social information converged on the correct response option, conformity should lead to an increase in participants' correct 2AFC performance, as compared to when the social information converges on the incorrect response option, and indeed this was the case (correct social information, 2AFC performance 85.2% (SD = 10.5) versus incorrect social information, 2AFC performance 57.3% (SD = 19.3), t(101) = 12.11, p<0.0001). Performance in these conditions also indicates that the combined influence of agreement from both partners over the participant's responses was much stronger than that observed in the conflicting social information conditions. When both partners gave correct information, the participants mean correct 2AFC rate was significantly higher than when only the similar partner was correct (85.2% versus 76.6%, t(101) = 6.14, p<0.001). When both partners gave incorrect information, the participants mean correct 2AFC rate was significantly lower than the condition where only the similar partner was incorrect (57.3% versus 70.1%, t(101) = 7.08, p<0.001).

## Discussion

We quantified the extent to which participants explicitly simulated their partners' beliefs based on their own beliefs. We observed that participants only simulated at levels above chance when they inferred the beliefs of the partner who had frequently agreed with their own beliefs during the prior exposure phase. In marked contrast, we could find no quantifiable evidence for simulation of the less agreeable, hence more dissimilar, partner. It is worth while noting that this could have emerged either in the form of an above or a below chance mirror score for the dissimilar partner, and that a below chance mirror score would indicate that the participants employed the opposite of their own opinion [21] [22]. Instead, however, we observed a mean dissimilar partner mirror score that did not differ from chance.

Having established that belief-simulation was restricted to the similar partner, was this difference in mentalizing associated with a subsequent bias in conformity towards the similar partner's memory during the collaborative memory task? The 2AFC results clearly indicated that this was the case. When faced with different information from each partner, participants chose the information provided by their similar partner significantly more often than that provided by their dissimilar partner. This led to a pattern of relatively increased 2AFC accuracy when the similar partner endorsed the correct versus incorrect response option. These findings suggest that belief-simulation leads to a systematic bias in memory conformity towards similar and away from dissimilar individuals, in the absence of any objective evidence that the simulated individual has a more accurate memory.

Our findings appear to reveal a novel systematic bias in the expression of memory conformity towards like-minded individuals and away from individuals who do not share our beliefs. These new findings follow in a long tradition of work, stretching at least as far back as the 1950s [24], examining social and interpersonal influences over individual cognition. More recently, there has been a surge in research dealing with such influences on memory from a forensic perspective. This perspective views other people's memories as a pernicious corrupting influence that is highly undesirable. Such influences can distort memory accuracy and generate spurious patterns of corroboration between individuals that plague criminal justice systems all over the world. It seems, however, that the willingness to engage with another person's memory may also be interpreted as a highly adaptive reaction to a rich source of information about the world we and other people inhabit. Similarly, the benefits of social, or group learning, are also emphasized in work on the added value of learning with other people as opposed to on one's own [25] [26]. The potential benefits of such social learning do not come without potential costs, however, and so it is highly likely that evolution will have selected strategies for social learning that promote its advantages and offset its costs [1] [19].

Frith and Frith [1] emphasize two kinds of benefit associated with social learning, from an adaptive perspective. One is improved accuracy within an individuals' own mental model of the world, that may in turn enhance their decision-making abilities. The second benefit acts indirectly by enhancing mutual trust and promoting our ability to flexibly cooperate with one another. To function effectively in both these ways requires us to apply meta-cognition to other people or, in other words, to mentalize [1] [27]. In this paper we have focused on the idea that explicit mentalizing mechanisms play an adaptive role during social encounters that involve sharing knowledge about the past. We have argued that substantial support for the adaptive perspective is already available from studies of memory conformity originating from the forensic perspective [3]. We briefly reviewed this evidence in the introduction, which led us to the conclusion that memory conformity is regulated by insight into factors that modulate memory accuracy in similar ways in one's self and other people. People, quite reasonably, use their own memory function as a mirror to regulate when and to whom they should conform.

In the new experiment that we report, we examined whether belief-simulation also functionally biases the social partners that we choose to conform to. Our findings revealed a pattern consistent with a link between increased belief simulation and enhanced conformity. The findings indicate that engaging in belief-simulation about another individual enhances their relative social influence over our memory. Hence, projecting our own beliefs onto others makes us more likely to endorse what such individuals remember, and less likely to endorse what dissimilar individuals remember. Our findings therefore compliment recent work from Echterhoff and colleagues [23] [28] who have examined social influences over memory as a function of other people's in-group/out-group status. Their work has revealed that people are more willing to adopt and incorporate information about the past from in-group versus out-group members, where this categorization is based on easily observable cues such as age, gender or race.

For example, Lindner et al. [23] recently showed that the increased social influence of in-group members' memory leads to increased self versus other confusion over who performed specific actions. These source-monitoring errors are enhanced when actions are performed during encoding by an in-group versus out-group member. Lindner et al proposed (and see [29]) that this confusion may have resulted from increased motor-simulation while viewing acts performed by in-group versus out-group members, a conclusion they based on findings from social neuroscience studies of the mirror neuron system [30] [31]. The present findings compliment this work by showing that engagement of belief-simulation also modulates social influence over memory.

Our experiment demonstrates a conformity bias that appears to operate independently of objective differences in the accuracy of a social partner's memory. We make this conclusion, while fully acknowledging that participants could have falsely and systematically believed that their similar partner had a more accurate memory than the dissimilar partner. It is not straightforward to generate an account as to why the mentalizing task would have led to this mistaken view of the social partners' memories, but the available data does not allow us to rule out the possibility. What seems more likely, however, is that belief-simulation triggered an enhanced normative social influence [32]. Our present findings therefore add to studies of such influence, which have found that a close personal relationship enhances the tendency to conform to another person [12] [33]. Our findings suggest, however, that it may be possible to bypass the need to recruit participants who have complex pre-existing relationships and instead another person's social proximity may be controlled and manipulated using the opinion statements, and the mentalizing task.

It therefore seems reasonable to us that simulation may have altered our participants' normative relationship with their partners, without altering the participant's views about the likely accuracy of their partners' memory. But this claim needs to be tested in future work and, in closing, we would like to suggest three possible directions for such studies (we thank an anonymous reviewer for drawing our attention to these three distinct lines of work). First, it would seem useful to examine the scope of the enhancement in trust that simulation generates. It may be that simulation enhances trust in various contexts, not just those involving trust in what a partner remembers about the past. For example, simulation may enhance trust in what a partner has to say about events in the future. Second, studies are needed to investigate whether simulation may in fact alter the way that one evaluates the quality of another person's knowledge. For example, are there circumstances where simulation leads one to over-value information from certain individuals, even if one has reason to believe that their memory is likely to be poor? Third, the effects of simulation on normative influence should be explored under conditions where such influences are heightened. For example, by employing live interactions between individuals, rather than virtual partners. A key issue here would be to determine whether simulation under such conditions tends to enhance conformity, or if there are circumstances where conformity may be enhanced to dissimilar partners, for example to offset any negative evaluation of one's self by such partners.

## Conclusions

We have argued that the adaptive perspective provides a more fundamental perspective upon the phenomenon of memory conformity, in comparison to the forensic perspective that has guided research in recent years. The adaptive perspective encourages greater attention to the social cognitive mechanisms that bias when and to whom we conform, complementing the forensic perspective which is more focused upon the consequences of conformity for the accuracy and independence of an individual's memory. We ran a novel experiment showing, for the first time to our knowledge, that trust in other people's memory is modulated by the extent to which we project our beliefs onto them, even when there is no objective evidence that simulated individuals are more accurate than dissimilar individuals towards whom we do not project our own beliefs. Alongside recent work originating from the forensic perspective, our new findings strongly suggest that conformity is not merely a sign of weak or dysfunctional memory, but is instead an expression of explicit mentalizing mechanisms that work adaptively to enhance the accuracy of our mental representations, and our trust in like-minded individuals with whom we may cooperate for mutual benefit.
